# Quantitative Investigation of Hand Grasp Functionality: Hand Joint Motion Correlation, Independence, and Grasping Behavior

**DOI:** 10.1155/2021/2787832

**Published:** 2021-12-02

**Authors:** Yuan Liu, Bo Zeng, Ting Zhang, Li Jiang, Hong Liu, Dong Ming

**Affiliations:** ^1^Tianjin University, Academy of Medical Engineering and Translational Medicine (AMT), Tianjin, China; ^2^Beijing Institute of Precision Mechatronics and Controls, Laboratory of Aerospace Servo Actuation and Transmission, Beijing, China; ^3^Soochow University, College of Mechanical and Electrical Engineering, Robotics and Microsystems Center, Suzhou, China; ^4^Harbin Institute of Technology, State Key Laboratory of Robotics and System, Harbin, China

## Abstract

Modeling and understanding human grasp functionality are fundamental in prosthetics, robotics, medicine, and rehabilitation, since they contribute to exploring motor control mechanism, evaluating grasp function, and designing and controlling prosthetic hands or exoskeletons. However, there are still limitations in providing a comprehensive and quantitative understanding of hand grasp functionality. After simultaneously considering three significant and essential influence factors in daily grasping contained relative position, object shape, and size, this paper presents the tolerance grasping to provide a more comprehensive understanding of human grasp functionality. The results of joint angle distribution and variance explained by PCs supported that tolerance grasping can represent hand grasp functionality more comprehensively. Four synergies are found and account for 93% ± 1.5% of the overall variance. The ANOVA confirmed that there was no significant individual difference in the first four postural synergies. The common patterns of grasping behavior were found and characterized by the mean value of postural synergy across 10 subjects. The independence analysis demonstrates that the tolerance grasping results highly correlate with unstructured natural grasping and more accurately correspond to cortical representation size of finger movement. The potential for exploring the neuromuscular control mechanism of human grasping is discussed. The analysis of hand grasp characteristics that contained joint angle distribution, correlation, independence, and postural synergies, presented here, should be more representative to provide a more comprehensive understanding of hand grasp functionality.

## 1. Background

Tremendous grasp functionality is one of the critical characteristics of humans [1–4]. More than 20 degrees of freedom (DoFs) [5–7] are coordinately actuated by multiple extrinsic and intrinsic muscles [8] and controlled by a huge amount of neural resources [9]. Any bone, muscle, and nerve damage often leads to impairment of hand movement, which will seriously affect the quality of life [10], such as stroke, Parkinson's disease (PD), or physical injury. All of these critical particularities of the human hand have attracted investigators to study hand grasp functionality. People want to understand human hand grasp functionality as comprehensively as possible. In neuroscience, investigators expect to establish experimental paradigms that can effectively represent human grasp functionality in order to explore the neural mechanism of motor control. In rehabilitation, the therapists hope that they can more comprehensively and accurately evaluate the hand grasp function for clarifying the functionality impairment to formulate the personalized treatment plans. In robotics, roboticists expect to comprehensively understand grasp functionality and characteristics in order to design and control prosthetic hands or exoskeletons to help patients reconstruct the hand grasp functions.

The systematic research of hand grasp function starts from the functionality assessment in rehabilitation [11]. A discrete set of grasp types is used to qualitatively summarize hand grasp function. Along this direction, a number of researches have investigated the classification of human grasp, as shown in Table 1. Schlesinger [11] classified human grasp to 6 typical postures according to the object shape, while Napier [12] found that people may grasp the same object with different postures according to different aim goals (power and precision grasp). Kamakura et al. [13] added the intermediate grasp to supplement aim goals. The 14 typical grasp postures were used to describe the hand grasp functionality. Iberall and Iberall and Bingham [14–16] classified hand grasp posture based on the oppositional force consideration. By summarizing the related researches, 25 typical postures were used to describe human grasp functionality. Consequently, three main classification definitions are obtained: object shape, aim goal, and oppositional force and virtual finger. In this case, after synthetically considering the three classification definitions, Cutkosky [17], Stival et al. [18, 19], and Feix et al. [20] proposed their grasp taxonomies using 16, 20, and 33 typical grasp postures to represent human grasp functionality, respectively.

In neuroscience, human grasp kinematics is extensively investigated to quantitatively discover the effects of particular factors on human reach-to-grasp process or a particular behavioral phenomenon in a specific context, as shown in Table 2. Jeannerod proposed the Dual Visuomotor Channel theory [21] to lay the foundation for the related studies: human prehension actually consists of two distinct but temporally integrated movements, a reach and a grasp. Reach and grasp are mediated as a function of extrinsic object properties (e.g., object position and orientation) and intrinsic object properties (e.g., object size and shape), respectively. Along this direction, several particular intrinsic and extrinsic object property effects on reach-to-grasp are investigated. Most of them only focus on the single factor, as shown in Table 2, such as the intrinsic object properties (e.g., size [22], fragility [23], texture [24], and mass [25, 26]), extrinsic object properties (e.g., initial position [27] and target position [28, 29]). The studies of the particular context are difficult to comprehensively represent human grasp functionality. In addition, these studies mainly focus on the process of the entire arm movement of reach-to-grasp process, rather than the hand-centric consideration. The measures mainly contained hand movement time, grasp aperture, and finger (hand) position on objects (Table 2), rather than the detailed grasp posture.

A portion of studies focuses on hand kinematic synergies in some specific contexts, as shown in Table 3. Santello et al. [30] investigated the hand postural synergies in 57 imagined object grasping. Mason et al. [31] studied the reach-to-grasp synergies of 16 columnar object grasping. Hereafter, the kinematic synergies are investigated in different application considerations and grasp conditions (e.g., biometrics for secure identity verification [32], precision grasp for cylinder of different size [33], haptic exploration [34], rapid grasping [35], and bimanual manipulation [36]). Different objects (8 to 57 types in Table 3) are selected to span the grasp functionality in the corresponding specific context. However, these studies focus on some particular application considerations and grasp conditions, rather than a general and representative understanding of human grasp functionality.

On the other hand, the investigators attempt to understand human nature grasping behavior through behavior recording in unstructured environment. Bullock et al. [37–40] built a video dataset collected from a head-mounted camera to record hand usage in two housekeepers and two machinist work activities. Ingram et al. [41] use Cyberglove to track the hand movements of six subjects outside of a laboratory setting. Unstructured environment grasping provides a paradigm to understand human general grasp behavior. However, due to the randomness of grasping context, it is difficult to help people quantitatively understand the detailed grasp postures Moreover, the rigorous conditions of unstructured environments outside the laboratory limit the use of large medical imaging equipment (e.g., fMRI and MEG) and motion capture equipment. It is difficult to be used to explore the neuromuscular control mechanism of human grasping. Based on the large medical imaging equipment (e.g., CT and fMRI), numerical hand models have been well-developed to investigate hand grasping and biomechanics. Wei et al. have developed a subject-specific finite element human hand model to study hand grasping in 2019 [42].

Consequently, there are several open points in the literatures for providing a comprehensive and quantitative understanding of human grasp functionality. Firstly, the studies of grasp classification mainly focus on a few typical grasp types on the basis of the qualitative grasp classification definition. It is difficult to understand human grasp functionality comprehensively and quantitatively, but the classification definition can help us complete the representative influence factors of human grasp. Secondly, for the grasp kinematic studies, the objective is to discover the particular influence factors on human reach-to-grasp or a particular behavioral phenomenon in a specific context. Besides, these studies focus on the movement functionality of the entire upper limb including the hand, wrist, and arm rather than the hand-centric consideration in detail. Therefore, the related studies have difficulty providing a comprehensive understanding of human grasp functionality. Thirdly, the studies of hand kinematic synergies are also investigated in some particular application considerations and grasp conditions. The results make it difficult to comprehensively represent human grasp functionality. Fourthly, for human natural grasp in unstructured environment, it is difficult to help people quantitatively and parametrically understand human grasp behavior in detail. The rigorous conditions outside the laboratory limit the use of large medical imaging equipment to explore the neuromuscular control mechanism of human grasping.

Different from previous studies, we attempt to complete the significant influence factors on human grasping, integrate them to establish a laboratory-based unstructured experimental paradigm to efficiently represent human general grasp functionality, and further improve the application range, such as neuroscience, rehabilitation, and robotics. In an accompanying study [43], we demonstrate that the relative position between the human hand and object, as a general and essential influence factor in daily grasping, can cover the main definitions of grasp classification and seems a direct influence factor and indicator of grasp planning parameterization. The relative position seems another important basic influence factor that significantly affects human grasping in addition to the object size and shape, which is seldom investigated in previous researches. Therefore, we simultaneously considered the three general influence factors that contained relative position, object shape, and size in this paper in order to provide a comprehensive understanding of human grasp functionality.

In this paper, we quantitatively investigate the human tolerance grasping (human can successfully grasp various objects in different acceptable relative positions between the human hand and object). The object shape (sphere, cylinder, and prism), size (small and large), and relative positions (*X*/*Y*/*Z* deviations) between the human hand and object are all considered as a whole in order to represent human grasp functionality more comprehensively. On this basis, joint angle distribution and variance explained by PC results supported that tolerance grasping can represent hand grasp functionality more comprehensively. Four synergies are found and account for 93% ± 1.5% of the overall variance. The ANOVA confirmed that there was no significant difference in the first four postural synergies across 10 subjects. Therefore, the common pattern of grasping behavior was found and characterized by the mean value of postural synergy across 10 subjects. The independence analysis demonstrates that the tolerance grasping has potential advantages to explore more accurate representation of hand neuromuscular architecture and control mechanism.

## 2. Methods

**Figure 1:**
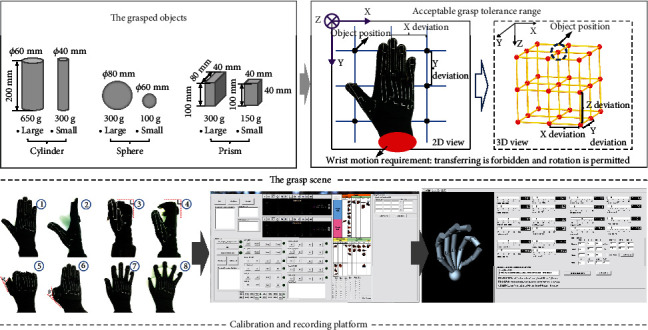
The grasped objects, acceptable range, Cyberglove calibration, and recording platform.

The same experimental setup (the view of experiment, the grasping tolerance determination process), protocol (experiment requirements), dataset, and calibration (Cyberglove calibration process) as in the accompanying paper [43] and a recent study [44] were used. The concise description about the grasped object shapes and sizes, tolerance range, posture calibration, and recording platform is shown in Figure 1. The object shape, size, and weight of objects we chose were based on the Feix et al. [39], Zheng et al. [45], and Bullock et al. [38, 46] research results to high-effectively represent the objects we grasped in daily life. The static grasping postures that can lift up the object are recorded, and the posture that cannot lift up the object is eliminated until the stable grasping is completed to record the corresponding posture. Two repeats are needed. Thus, totally, 3240 postures (10 subjects × 6 objects × 27 relative distances × 2repeats) are collected in posture dataset. Hand grasp posture that contained 15 joint information was actually recorded by Cyberglove III (Virtual Technologies, Palo Alto, CA) at a resolution of <0.1° and sampled at 100 Hz each. The following joint angles were measured: proximal interphalangeal (PIP) joints and metacarpophalangeal (MCP) joints of digits II-V, as well as the interphalangeal (IP) and MCP joints of the thumb (digit I), opposition rotation (Rot) of the thumb, abduction/adduction (ABD) of the thumb carpometacarpal (CMC) joint, and MCP joints of digits II–V. The step (2) figure of the glove calibration process figure in Figure 1 shows the opposition rotation of thumb Rot joint. The step (3) and (4) figures show the flexion of four finger MCP and PIP joints, respectively. The step (5) and (6) figures show the flexion of thumb IP and MCP joints, respectively. The step (8) figure shows the abduction of ABD joints. The dataset is based on 10 healthy subjects (24~27 years old, 8 men and 2 women) grasping six typical objects (3 shape × 2 size) in 27 relative positions (3 *X* deviation × 3 *Y* deviation × 3 *Z* deviation) within acceptable range which was obtained in preexperiment. Within the grasp tolerance range, subjects can successfully grasp objects.

### 2.1. Basic Analysis

As the first step of data analysis, in order to basically understanding the distribution of each joint angle across ten subjects, two repeats in a total of 3240 postures were averaged and a general matrix was created containing all subjects' motion data; then, the general matrix is decomposed to ten subject matrixes corresponding to each subject. The rows of general matrix represent different grasp conditions of 10 subjects, 1620 rows in total (10 subjects × 6 objects × 27 relative positions), while the rows of subject matrix represent different grasp conditions of the corresponding subject, 162 rows in total (6 objects × 27 relative positions). Each joint angle was set in the corresponding columns, 15 columns in total (4 for thumb (Rot, MCP, IP, and T-ABD), 2 for each finger (MCP, PIP) × 4 fingers, and 3 for ABD between adjacent fingers (I-M/M-R/R-P ABD)). The mean of each joint angle, maximum (Max), minimum (Min), and motion range (Range) of each subject are calculated based on each subject matrix, then all averaged across ten subjects to represent the general characteristics of each joint angle. The skewness of each joint angle distribution is presented based on the general matrix for understanding the general distribution of all ten subject joint angles.

### 2.2. Correlation Analysis

The correlation analysis is implemented in each two columns of general matrix; then, the absolute values of the correlation coefficients are obtained and arranged in the general correlation matrix. The column and row both represent joint types in the order of thumb (Rot, MCP, IP, and T-ABD), MCP of four fingers (I, M, R, and P), PIP of four fingers (I, M, R, and P), and ABD joints between four fingers (I-M, M-R, and R-P). The boxplots are used to understand the general distribution of absolute values to the correlation coefficients between joints, which shows that the distribution to each joint is relatively scattered. For further investigating motion correlations of finger and joint type, the joints are arranged into the finger (T, I, M, R, and P) and joint type (MCP, PIP, and ABD) unit. Correlations between each joint in the unit and the other joints were calculated and averaged. In this case, the finger and joint type movement correlations are shown. For the finger unit, the thumb unit contained the corresponding Rot, MCP, IP, and T-I ABD, while for the other four finger units (I, M, R, and P), each finger unit contains the corresponding MCP and PIP joints. For the joint type unit, MCP and PIP unit contained four-finger MCP and PIP joints, respectively, while the ABD unit contained four ABD joints between each two adjacent fingers. According to the boxplot results of each joint, we find that there is no obvious difference to the mean and median of different joints. However, the maximum correlation coefficients to the joints in each finger can be clearly discriminated, especially for thumb and four fingers. Thus, we conducted statistics on the maximum correlation coefficient to each joint of each subject. Taking fingers as a unit, the maximum correlation coefficients and the corresponding paired joints of each given finger to 10 subjects are analyzed to show the individual differences of finger movement correlations. As such, if the differences are not obvious, for example, the results of 10 subjects are completely uniform, it indicates that the given finger motion may completely be restricted by the inherent constraint in musculoskeletal and neuromuscular architecture, and shows the uniform motion mode. Conversely, if the differences are obvious, it indicates that the given finger motion is more dexterous with lots of motion modes that varied in different subjects, and the corresponding motion are more complex.

### 2.3. Finger Independence Measure

Finger motion independence is the reflection of hand neural and neuromuscular architecture, which has been explored extensively [41], [47, 48]. We selected two representative investigations that contained the human natural hand movement in unstructured environment [41] and cortical sites encoding for each finger movement [9], which are used to compare with our results for inspecting whether human tolerance grasping has the potentials in exploring neuromuscular control mechanism of human natural grasping in laboratory.

Inspired by the previous study [41], in order to quantify the finger independence, we use the linear regression to fit each joint motion of the given finger as a function of the joint motion of other fingers and ABD module. We calculated the percentage of the finger's movements that were unexplained by this linear fit. More specifically, we calculated the ratio of the variance to the residual of the linear reconstruction to the total variance for the corresponding finger. This yielded the unexplained variance percentage to each finger for quantifying the independence of each digit's movements. As such, a value of 0% would indicate that the movements of the particular finger could be completely predicted by linear reconstruction using the movements of the other four fingers. Conversely, a value of 100% would indicate that none of the movements of a particular finger could be predicted from the other four fingers.

The thumb contained four joints: opposition rotation (Rot) and the flexion/extension of MCP, IP, and T-ABD joint; each finger of four fingers contained two joints: the flexion/extension of MCP and PIP joint. As the ABD joints in Cyberglove are set in the adjacent fingers, they cannot correspond to each single finger and are divided into a single ABD module. ABD joint module does not exist as a dependent variable in linear regression. The general matrix is divided into ten subject matrixes corresponding to each subject. The columns also represent the joint types the same as the general matrix, while the rows only represent the grasp conditions to the single subject and a total of 162 rows (6 objects × 27 relative distances). The finger independence is calculated in each subject matrix. In this case, the subject mean and standard error (SE) are presented.

### 2.4. Postural Synergy Analysis

The principal component analysis (PCA) is implemented to each subject grasp data and normalized to allow intersubject comparisons of hand motion patterns. The postural synergies are used to efficiently represent the hand movement characteristics. One-way ANOVA was performed to further quantitatively test individual differences of hand movement characteristics. Independent factors were subject (1-10). The dependent variables were four principal components.

## 3. Results

### 3.1. Basic Statistics

The first analysis step is to basically understand the joint angle distribution, as shown in Table 4. The mean posture performs like a neutral position (hand in a relaxed natural state so that the joints are minimizing stress on the tendons, muscles, and skeletal system) in our daily lives. The standard deviation indicates the dispersion degrees near the mean value of each joint movement. The thumb performs a large dispersion. The movement is more complex, especially for the opposition rotation (T-Rot) and interphalangeal (T-IP) joint (the only two joints in which SD is larger than 30). The maximum (Max), minimum (Min), standard deviation (SD), and movement range (Range) to the most of joints in tolerance grasping are generally larger than human natural grasp [41] and some motion range investigation studies [49], especially for thumb Rot and MCP and ABD between fingers (the extreme joint angles induced by extreme gestures are shown in the Electronic Supplementary Material (available here)), which indicates that the tolerance grasping induces more grasping postures that do not occur in nature grasping due to the extreme condition. This will help reflect the hand grasp ability more comprehensively, especially for understanding the musculoskeletal architecture and neural control mechanism of the human hand. The skew of the angle distribution indicates that in different grasp postures, whether the joint is more often flexed (negative skew) or more often extended (positive skew), the thumb, index, and middle fingers are more often flexed, while the ringer finger and pinky are more often extended. Thumb ABD (T-ABD) is more often adducted (positive skew).

### 3.2. Motion Correlation Analysis

**Figure 2:**
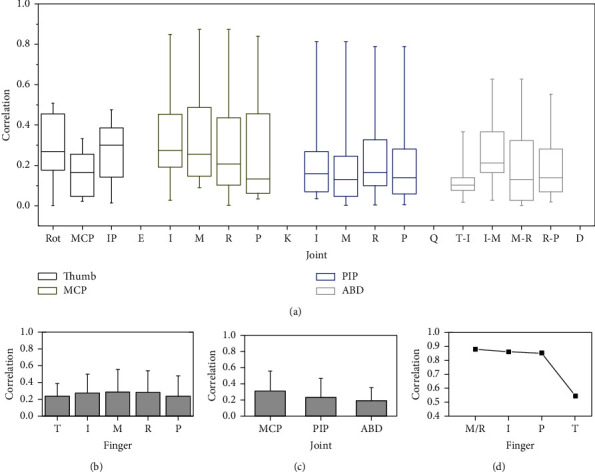
Correlation analysis of each joint movement for all subject data totally: (a) distribution of each joint correlation, (b) finger movement correlation, (c) joint movement correlation, and (d) the highest correlation to finger/joint. T, I, M, R, and P represent the thumb, index, middle, ring finger, and pinky; T-I, I-M, M-R, and R-P represent the ABD joint between the adjacent fingers from the thumb-index finger to ring finger-pinky.

For the second analysis step, the boxplots show the data distribution in 4 segments between the minimum, the first quartile, median, third quartile, and the maximum, which is used to show the correlation coefficient distribution to each joint for all 10 subjects, as shown in Figure 2(a). It can be seen from the figure that the distribution is relatively scattered and medians to the absolute values of the correlation coefficients of each joint are near 0.2. No obvious difference was shown between different joints. In addition, taking the finger or joint types as the unit, we calculated the mean correlation coefficients based on the general correlation matrix, as shown in Figures 2(b) and 2(c). Similar to the medians in Figure 2(a), no obvious difference was shown to different fingers and joint types.

As shown in Figure 2(a), the correlation coefficient distribution of the four-finger MCP and PIP joints is obviously more scattered, due to the obviously larger value of the maximum correlation coefficients, which shows that the obvious difference occurred across the four fingers, thumb, and ABD joints. Therefore, the results indicate that the maximum correlation coefficients have a higher statistical significance compared with the mean and median. In fact, the maximum correlation coefficient is also more practical, such as for inspiring the mechanical design and control of the robotic hand. In this case, taking the finger as the unit, the maximum correlation coefficient of each finger that contained joints is shown in Figure 2(d), which shows that the thumb maximum correlation coefficient is significantly lower than the other four fingers.

**Figure 3:**
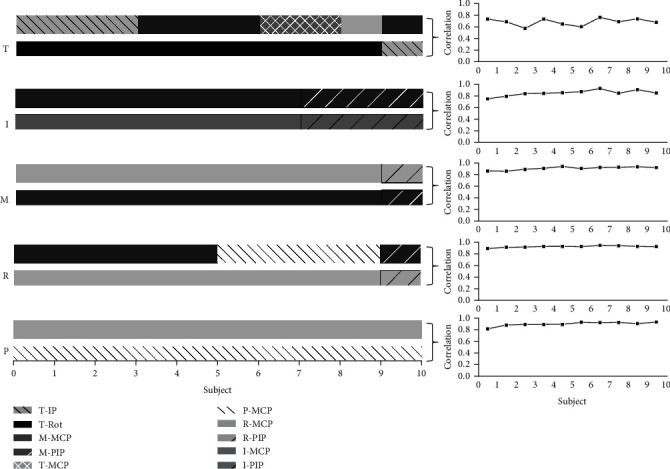
Paired joints with the highest correlation coefficients in each finger for each subject and the highest correlation coefficients in each finger across ten subjects.

In order to clearly clarify the individual differences for the joint pair of the highest correlation, taking the finger as the unit, we investigated the joint pair of the highest correlation in each particular finger, as shown in Figure 3. The individual differences reflect the individual grasp habits. The larger individual differences mean the finger movement is less limited by the hand inherent neuromuscular architecture. The finger movement is more complex. Higher consistency means the general grasp habits and limitation of the inherent neuromuscular architecture. It can be seen from Figure 3 that the consistency of the thumb was the lowest. The 10 subjects showed 5 different joint pairs. The thumb side joint in each joint pair is more consistent. Nine of ten subjects showed the opposition rotation joint. The corresponding joints are mainly distributed in MCP joints of the middle and ring finger. This indicates that the thumb often performs the opposition rotation with the palm and fingers when the middle and ring fingers are flexed. In addition, the joint pair of the pinky, middle, and index finger performs a relative consistent distribution across ten subjects, which mainly shows the high movement correlation between adjacent fingers, while for ring finger, the highest correlation paired joints are approximately equally distributed in the middle finger (5 subjects) and pinky (4 subjects). It shows that ring finger movement generally performs the combined movement with middle finger and pinky.

Moreover, we find five high correlation joint pairs, which are expressed in the form of (*x*, *y*) and mean that the largest correlation joint with the *x* joint is all *y* joints for all ten subjects. These joint pairs showed complete consistency across 10 subjects, showing the high consistent motion correlation relationship. The five high correlation joint pairs are found as follows: (I-MCP, M-MCP), (M-MCP, R-MCP), (P-MCP, R-MCP), (I-PIP, M-PIP), and (P-PIP, R-PIP), shown in Table 5 of discussion part.

### 3.3. Finger Independence Analysis

**Figure 4:**
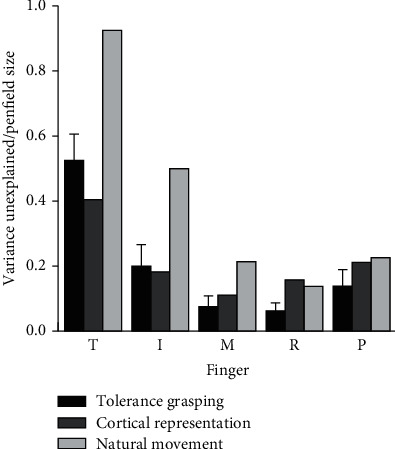
Analysis of the finger movement independence in tolerance grasping, cortical representation, and natural movement of unstructured environment. The cortical representation and natural movement in unstructured environment results are replotted from [9, 41].

**Figure 5:**
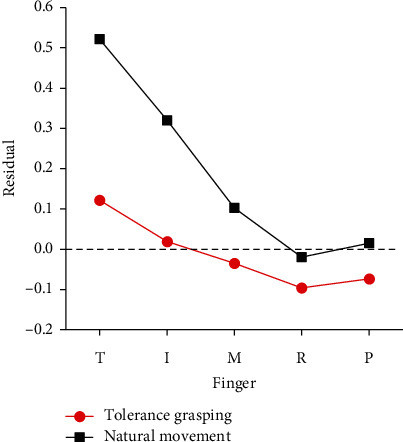
Residual according to Figure 3 results between cortical representation and tolerance grasp and natural movement in unstructured environment.

As shown in Figure 4, the vertical axis of variance unexplained is the independence metric in our research and the selected research of natural hand movement in unstructured environment [41], while the Penfield size represents the number of cortical sites encoding for each finger movements (from the only electrophysiology study of neuron populations corresponding to each finger movement [9]). It can be seen from the figure that tolerance grasping shows a similar distribution trends of finger independence with hand natural movement in unstructured environment and cortical representation of each finger movement. A parabola distribution with high on the two sides and low on the middle is presented. The thumb is the most independent digit, while the index finger is the most independent finger across four fingers, and the ring finger is the least independent digit. This is consistent with hand natural movement [41], and the results have a strong correlation (*r* = 0.98, S1_Fig of Electronic Supplementary Material (available here)) with the results from hand natural movement in the unstructured environment outside the laboratory. This may indicate that after considering the influence of object shape, size, and relative positions, the tolerance grasping can be seen as a laboratory-based unstructured environment that seems to be able to efficiently represent the unstructured natural grasping outside of a laboratory setting. In order to clarify the difference of the independence results between tolerance grasping and natural movement, the residuals between independence metrics of grasp conditions (tolerance grasping and natural movement) and cortical representation of finger movement according to Figure 4 are presented in Figure 5. It can be seen from the figure that the residuals of tolerance grasping are obviously smaller than those of natural movement, especially for the thumb, index, and middle finger.

### 3.4. Postural Synergy Analysis

**Figure 6:**
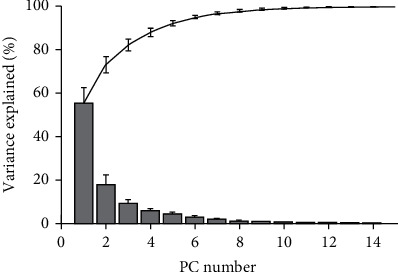
Information transmitted by PCs (subject mean and SE).

The PCA can estimate the dimensionality of hand movements.  Figure 6 shows that the first two postural synergies (PC1~PC2) can explain much of total posture variance (79% ± 4.6%), while for first four postural synergies (PC1~PC4), they can explain 93% ± 1.5%variance information, which indicates that tolerance grasping can be reconstructed accurately by the first four PCs. In our previous study of extracting on all subjects' data simultaneously [44], we demonstrated that the first two PCs of tolerance grasping can only explain the information less than 65%. This is obviously lower than other studies of hand kinematic synergies in Table 3 (~80% in grasp imagined objects [30], ~99% in reach-to-grasp for columnar objects [31], ~99% in precision grasping for cylinder of different size [33], ~70% in haptic exploration [34], ~80% in rapid grasping [35], and ~88% in bimanual manipulation [36]). This indicates that the amount and dimension of information in tolerance grasping is increased as the simultaneous consideration of relative position, object shape, and size. These results quantitatively demonstrated that the tolerance grasping can represent human grasp functionality more comprehensively.

**Figure 7:**
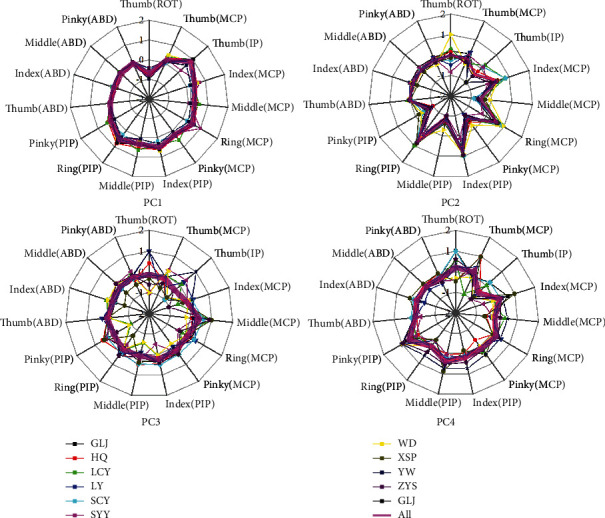
First four principal components of each subject. The principal components were normalized to allow intersubject comparison. The magenta thick lines in each subfigure represent the mean value of principal components across ten subjects.

**Figure 8:**
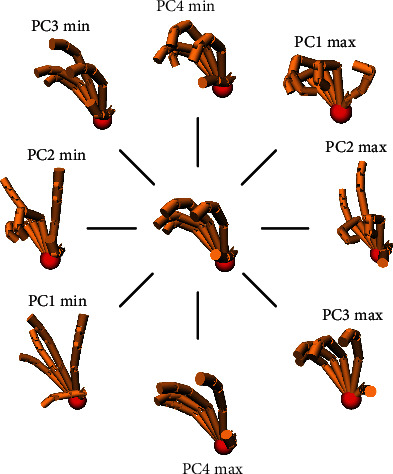
Movement characteristics along PC1~PC4.

Figure 7 shows the first four principal components of each subject. The principal components were normalized to allow intersubject comparison. It can be seen from the figure that PC1-PC4 keep the consistency across all ten subjects, especially for PC1 and PC2. The one-way ANOVA is performed to further quantitatively test individual differences, and the result shows that there was no significant difference in the postural synergy of the 10 subjects (*F* (9,140) = 0.49, *P* = 0.88 for PC1; *F* (9,140) = 0.62, *P* = 0.78 for PC2; *F* (9,140) = 1.22, *P* = 0.29 for PC3; and *F* (9,140) = 1.58, *P* = 0.13 for PC4). Therefore, the common pattern of grasping behavior was found and characterized by the mean value of postural synergy across 10 subjects, as shown in Figure 8. PC1 mainly reflects the similar degree of extension-flexion of four-finger MCP and PIP joints and the reverse motion between thumb Rot and IP joint, accounting for 55.3% ± 7.2% of the variance. PC2 explains 17.7% ± 4.6% of the variance and mainly reflects the reverse motion between adjacent fingers of four fingers and thumb IP joint motion. PC2 can reflect the finger independence motion and perform the posture diversity accompanying with other PCs. PC3 and PC4 explained 9.0% ± 1.9% and 5.7% ± 1.0% of the variance and mainly perform small range of motion to the five finger joints.

## 4. Discussion

The objective of this study is to provide a comprehensive and quantitative understanding of hand grasp functionality in detail and further clarify the applications in multiple areas, such as neuroscience, rehabilitation, and robotics. The results are only partially in accordance with previous studies, showing a similar distribution trend of finger independence and dimensionality reduction ability of kinematic synergies. However, the novel and general experimental paradigm, detailed statistical analysis, and novel results can contribute to a more comprehensive and much clearer clarification of human grasp functionality, which can facilitate applications in neuroscience, rehabilitation, and robotics.

The novelty of this study include the following: first, the relative position between the hand and object is given a particular attention (a general and essential influence factor in daily grasping, highly representative and direct indicator for understanding human grasp); second, comprehensive representation of hand grasp functionality (extensive investigations from multiareas, hand-centric consideration of three main influence factors, and number of grasp types); third, comprehensive understanding of hand grasp functionality, due to the detailed data analysis including basic statistics, motion correlation, independence, and postural synergy; fourth, the common pattern of grasping behavior was found and characterized by the mean value of postural synergy across 10 subjects; and fifth, a novel correlation analysis of hand joints (individual difference of the highest correlation joint pair in each particular finger).

For human grasp, we extensively investigated the related studies from grasp classification, human grasp kinematics, hand kinematic synergies, and unstructured grasping. These studies support to establish a general experiment paradigm for efficiently representing human grasp more comprehensively with a hand-centric consideration. In this study, we established a tolerance grasping paradigm. Three general influence factors of human daily grasping are simultaneously considered including relative position, object shape, and size in order to represent human grasp functionality more comprehensively. In terms of grasp type consideration, this study considered 3240 grasp types (10 subjects × 6 objects × 27 relative distances × 2repeats) performed by 10 subjects. Each subject performed 324 different grasp types (6 objects × 27 relative distances × 2repeats) that are much larger than 6~33 grasp types [11–19] (Table 1) of grasp classification studies and 9~57 object grasping of hand kinematic synergies [30–36] (Table 3) in order to provide a more comprehensive understanding of grasp functionality. In addition, the results of joint angle distribution and variance explained by PCs supported that tolerance grasping can represent hand grasp functionality more comprehensively. Firstly, the basic statistics indicates that the extreme condition of tolerance grasping induces more grasping postures that do not occur in nature grasping. The Max, Min, SD, and movement range of tolerance grasping are generally larger than natural movement [41]. Secondly, the postural synergy results indicate that the number of synergies that required explaining grasp variance is obviously larger than other studies [30–36], as the simultaneous consideration of relative position, object shape, and size.

The individual difference of the highest correlation paired joint can clarify the general finger and joint motion correlation across subjects. The results indicate that thumb opposition rotation correlates well with the flexion of the middle and ring finger, while ring finger movement generally performs the combined movement with the middle finger and pinky. In addition, five high correlation joint pairs are firstly found and showed complete consistency across 10 subjects with the highest correlation. Five joint pairs contained (P-PIP, R-PIP), (M-MCP, R-MCP), (P-MCP, R-MCP), (I-PIP, M-PIP), and (I-MCP, M-MCP). The five joint pairs are extensively distributed in MCP and PIP adjacent joints of four fingers, which is consistent with previous studies indicating the high correlation in MCP and PIP adjacent joints of four fingers [30, 41]. Four of them can be verified by our actual movement as shown in Table 5. When we try to independently flex the active motion joint within the coupling actuation module, the passive coupling joint will be coupling driven involuntarily.

The postural synergies are used to efficiently represent the hand movement characteristics. Figure 7 shows that PC1-PC4 keep the consistency across all ten subjects. The one-way ANOVA further verified that there was no significant difference in the postural synergy of the 10 subjects. Therefore, the common patterns of grasping behavior were found and characterized by the mean value of postural synergy across 10 subjects. Finger independence analysis results indicate that tolerance grasping has potential advantages in exploring the neuromuscular control mechanism of human grasping. Figure 4 indicates that tolerance grasping shows a similar distribution trends of finger independence with hand natural movement in unstructured environment and cortical representation of each finger movement. Figure 5 shows that the independence results of tolerance grasping are closer to cortical representation investigation results than those of natural movement, due to the fact that the variance of finger movements of natural grasp is significantly smaller than that of the tolerance grasping because fingers are in the rest state most of time in natural unstructured environment [41], which will increase the unexplained variance percentage of Figure 4. Moreover, the extreme condition of tolerance grasping can induce more grasp postures (the Max, Min, SD, and movement range of each joint are generally larger than natural movement [41]) that do not occur in nature grasping. These results indicate that the tolerance grasping can filter out the disturbance of the rest state and induce more grasp postures that contained the particular grasp in extreme condition. Therefore, tolerance grasping provides an efficient experimental paradigm that can more accurately represent hand neuromuscular architecture and control mechanism. Furthermore, the finger independence in tolerance grasping correlates well with cortical representation size of finger movement (*r* = 0.96, S2_Fig of Electronic Supplementary Material (available here)). All of these demonstrated that the tolerance grasp can help explore the neuromuscular control mechanism of hand grasp. Therefore, there are three potential advantages that the tolerance grasp is used to explore the hand neuromuscular control mechanism: (1) it can efficiently represent the unstructured natural grasping outside of a laboratory setting, which helps reveal the general neuromuscular control mechanisms rather than to each specific experimental scenarios; (2) the laboratory-based feature, which will facilitate the simultaneous use of large medical imaging equipment (e.g., fMRI and MEG) and motion capture equipment; and (3) more accurate representation of hand neuromuscular architecture and control mechanism.

## 5. Conclusion

In order to represent hand movement functionality more comprehensively, object shape, size, and relative positions are considered in our research. The results of basic analysis and variance explained by PCs supported that the tolerance grasping can represent human movement functionality more completely. Four synergies are found and account for >93% of the overall variance. The common pattern of grasping behavior was found and characterized by the mean value of postural synergy across 10 subjects. The independence analysis result shows the potential of tolerance grasping for exploring the more accurate neuromuscular control mechanism of human grasping. Both the literature survey and the experimental results in this paper support that the analysis results of tolerance grasping should be more representative to provide a more comprehensive understanding of hand grasp functionality. The analysis of this paper can serve many domains, such as neuromuscular control mechanism exploration, hand functionality rehabilitation, exoskeletons, prosthetic hand design and control, and packaging design of necessaries and products.

## Figures and Tables

**Table 1:** Summary of selected precious studies on grasp classification for describing hand grasp functionality.

Study	Total number of hand grasps	Classification definition	Description
Schlesinger [11]	6	Object shape	Six typical postures are proposed to describe hand grasp functionality according to the object shape.
Napier [12], Kamakura et al. [13]	14	Action goals (power, precision, intermediate grasp)	Power grasp holds object stably, while precision grasp imparts the object motion [12]. Intermediate grasp represents the postures with contact areas of finger-side aspect [13].
Iberall and Iberall and Bingham [14]-[16]	25	Oppositional force and virtual finger	For a given manual task, the grasp can be classified by the oppositional force exerted between virtual finger surfaces. Palm, pad, and side opposition means oppositional force along a direction perpendicular, parallel, and transverse to the palm, respectively.
Cutkosky [17]	16	Synthesis	Cutkosky [17] proposed a hierarchical tree of grasps, totally listing 16 different grasps. The grasp type, action goal, and VF oppositional force are synthetically considered.
Stival et al. [18], Jarque-Bou [19]	20	Synthesis	Stival et al. and Jarque-Bou et al. [18, 19] built a taxonomy that contained 20 grasp types. The grasp type, action goal, and VF oppositional force are synthetically considered.
Feix et al. [20]	33	Synthesis	Feix et al. [20] constructed a grasp taxonomy that contained 33 human grasp types. The grasp type, action goal, and VF oppositional force are synthetically considered.

**Table 2:** Summary of human grasp kinematic studies.

Study	Influence factor		Process	Measure
	Intrinsic object properties [31]	Extrinsic object properties [31]		
Bootsma et al. [22]	Size		Reach-to-grasp	Movement time, transport characteristics, grasp aperture
Savelsbergh et al. [23]	Fragility		Reach-to-grasp	Free-time duration and in-contact phase
Weir et al. [24]	Texture		Reach-to-grasp	Grasp aperture
Weir et al. [25]	Mass		Reach-to-grasp	Grasp aperture
Lukos et al. [26]	Center of mass location		Reach-to-grasp	Position of each digit tip at contact with the object
Armbrüster and Spijkers [27]		Initial position	Reach-to-grasp	Movement time and grasp aperture
Cohen and Rosenbaum [28]		Target position	Reach-to-grasp	Hand position (grasped height) on cylinder
Touvet et al. [29]	Shape, size	Target position	Reach-to-grasp	Grasp posture and hand position, orientation

**Table 3:** Summary of hand kinematic synergies in previous studies.

Study	Object types	Grasp condition	Process
Santello et al. [30]	57	Grasp imagined objects	Static grasp
Mason et al. [31]	16	Reach-to-grasp for columnar objects	Reach-to-grasp
Patel et al. [32]	25	Biometrics for secure identity verification	Reach-to-grasp and static grasp
Park et al. [33]	8	Precision grasp for cylinder of different sizes	Static grasp
Thakur et al. [34]	50	Haptic exploration	Reach-to-grasp
Vinjamuri et al. [35]	20	Rapid grasping	Reach-to-grasp
Jarrassé et al. [36]	9	Bimanual manipulation	Reach-to-grasp

**Table 4:** Basic statistics of the joint angle for all subjects.

Joints	Mean	SD	Max	Min	Range	Skewness
T-Rot	66	19	118	-19	137	-0.65
T-MCP	12	6	48	-25	73	0.10
T-IP	13	6	100	-12	112	-0.07
T-ABD	36	3	44	19	63	-1.7
I-MCP	29	8	80	-6	86	-0.10
I-PIP	41	13	81	-2	83	-0.14
M-MCP	29	6	89	-20	119	0.27
M-PIP	43	12	78	9	87	-0.36
R-MCP	37	6	92	-12	104	0.48
R-PIP	36	7	82	2	80	0.29
P-MCP	37	9	94	-6	100	0.42
P-PIP	41	13	91	4	87	0.17
I-M ABD	18	4	43	-5	48	0.34
M-R ABD	24	4	50	3	47	0.42
R-P ABD	17	4	37	2	35	0.17

**Table 5:** Verification of coupling actuation configuration.

Coupling actuation module	Motion scheme	Verification through the actual motion of our finger	
(P-PIP, R-PIP)	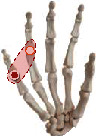	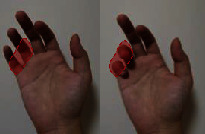	
(M-MCP, R-MCP)	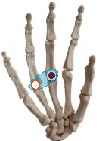	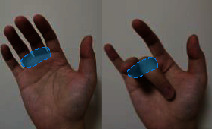	
(P-MCP, R-MCP)	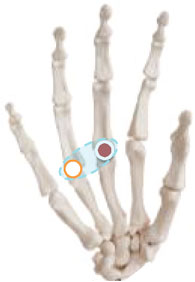	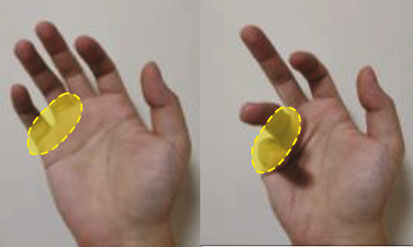	
(I-MCP, M-MCP)	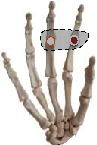	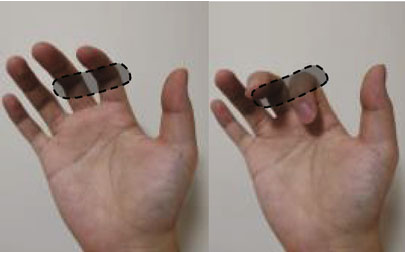	

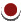	Active motion joint	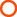	Passive coupling joint

## Data Availability

The datasets used and/or analyzed during the current study are available from the corresponding author on reasonable request.

## Supplementary Materials

Supplementary Materials Electronic Supplementary Material provides the supplementary explanation to the extreme joint angles induced by extreme gestures, and the supplementary figures provide the correlation analysis of the finger independence analysis results between hand natural movement and tolerance grasping and between tolerance grasping and cortical representation size of finger movement.
