# Supplementary material for: Quantitative Investigation of Hand Grasp Functionality: Hand Joint Motion Correlation, Independence, and Grasping Behavior
Source: Appl Bionics Biomech. 2021 Dec 2;2021:2787832. doi: 10.1155/2021/2787832 (PMC8660235; doi:10.1155/2021/2787832)
Supplement: Supplementary Materials — Electronic Supplementary Material provides the supplementary explanation to the extreme joint angles induced by extreme gestures, and the supplementary figures provide the correlation analysis of the finger independence analysis results between hand natural movement and tolerance grasping and between tolerance grasping and cortical representation size of finger movement. [file 2787832.f1.pdf]

Applied Bionics and Biomechanics

# Quantitative Investigation of Hand Grasp Functionality: Hand Joint Motion Correlation, Independence and Grasping Behavior

**(*Electronic supplementary material*)**

**Yuan Liu[1], Bo Zeng[2], Ting Zhang[3], Li Jiang[4], Hong Liu[4], Dong Ming[1]**

[1]Academy of Medical Engineering and Translational Medicine
Tianjin University
Tianjin, 300072, China.

[2]Beijing Institute of Precision Mechatronics and Controls
Laboratory of Aerospace Servo Actuation and Transmission
Beijing, 100000, China.

[3]Robotics and Microsystems Center
College of Mechanical and Electrical Engineering
Soochow University
Suzhou, 215000, China.

[4]State Key Laboratory of Robotics and System,
Harbin Institute of Technology,
Harbin, Heilongjiang 150080, China.

This document includes:

- Supplementary explanation to extreme joint angles induced by extreme gestures

- Supplementary figure

# Supplementary explanation to extreme joint angles induced by extreme gestures

Compared with the recent range investigation study [Ref 1] and natural grasp [Ref 2], most of joints in tolerance grasping are generally larger, especially for thumb Rot and MCP, ABD between fingers. Therefore, the author performed some extreme postures to reflect the extreme joint angles contain thumb Rot and MCP, and the abduction-adduction between adjacent fingers, as shown in S_Table1.

S_Table1 The extreme joint angles contain thumb Rot and MCP, and the abduction-adduction between adjacent fingers

| | | |
|---|---|---|
| Thumb Rot | 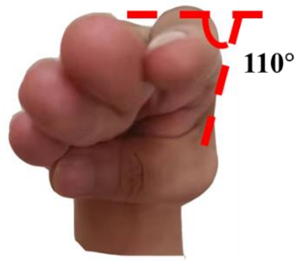 | 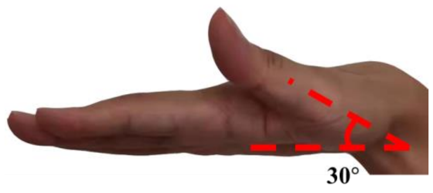 |
| Thumb MCP | 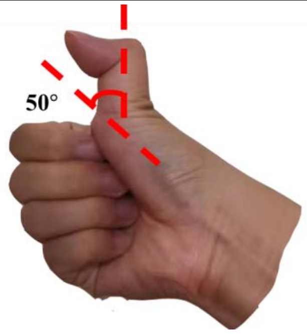 | 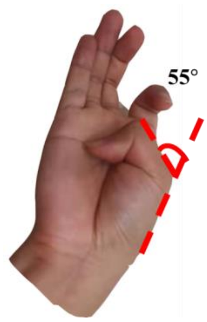 |
| Abduction-adduction between adjacent fingers | 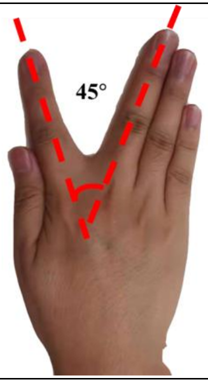 | 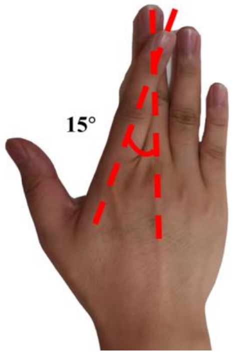 |

# Supplementary figures

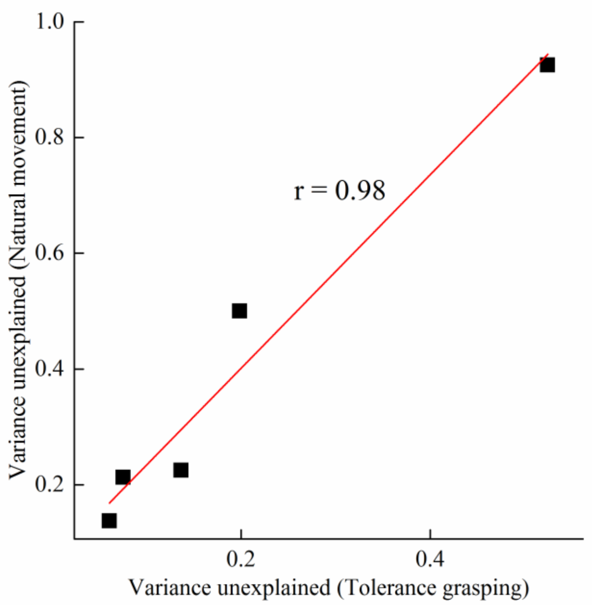


**S1_Fig.** The correlation analysis of the finger independence analysis results between hand natural movement [Ref 2] and tolerance grasping.

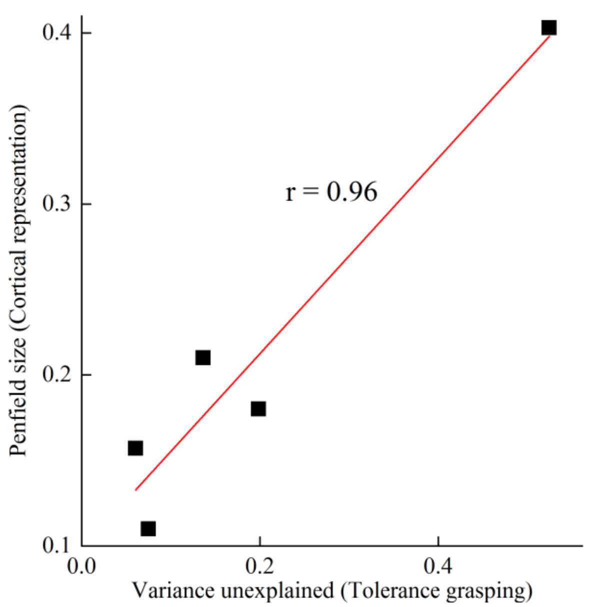


**S2_Fig.** The correlation analysis of the finger independence analysis results of tolerance grasping and cortical representation size of finger movement [Ref 3].

# Supplementary references

[Ref 1] Gracia-Ibáñez V, Vergara M, Sancho-Bru J L, et al. Functional range of motion of the hand joints in activities of the International Classification of Functioning, Disability and Health[J]. Journal of Hand Therapy, 2017, 30(3): 337-347.

[Ref 2] J. N. Ingram, K. P. Körding, I. S. Howard, and D. M. J. E. b. r. Wolpert. The statistics of natural hand movements. Experimental brain research, vol. 188, no. 2, pp. 223-236, 2008.

[Ref 3] W. Penfield and E. J. B. Boldrey, "Somatic motor and sensory representation in the cerebral cortex of man as studied by electrical stimulation," Brain, vol. 60, no. 4, pp. 389-443, 1937.
